# Anchorage‐independent and faster growth in clonal population from UV‐irradiated NER‐deficient cells

**DOI:** 10.1002/2211-5463.70195

**Published:** 2026-04-16

**Authors:** Paola Perucca, Anna Tricarico, Martina Furfaro, Priyam Ghosh, Ennio Prosperi, Lucia Anna Stivala, Ornella Cazzalini

**Affiliations:** ^1^ Dipartimento di Medicina Molecolare Università degli studi di Pavia Pavia Italy; ^2^ Istituto di Genetica Molecolare Luigi Luca Cavalli‐Sforza Consiglio Nazionale Delle Ricerche (CNR) Pavia Italy

**Keywords:** cancer, DDB2, drug resistance, proliferation

## Abstract

Human tumours are characterized by both molecular and functional heterogeneity, which drives cancer cell growth. In this context, the selection of subclones that acquire enhanced proliferative capacity and resistance to pharmacological treatments represents a necessary step in tumour progression. In the presence of DNA damage, efficient signalling and lesion removal are crucial in preventing the accumulation of genomic mutations. In Nucleotide Excision Repair (NER) pathways, the loss of interaction between DDB2 and PCNA (referred to as DDB2^PCNA‐^) results in genomic instability, delayed damage removal and activation of the epithelial‐to‐mesenchymal transition (EMT) transcriptional program. In this study, we demonstrate that human cellular clones, derived from UV‐damaged cells expressing DDB2^PCNA‐^, exhibit behaviours typically associated with tumour heterogeneity. Specifically, these clones show anchorage‐independent growth, differences in cell cycle regulation, frequency of atypical mitoses, expression of oncogenes and tumour suppressors and resistance to cisplatin treatment. Taken together, these findings highlight the critical role of DDB2‐PCNA interaction in maintaining genome stability and preventing cellular transformation.

## Graphical abstract

UV‐irradiated cells expressing a DDB2 mutant protein unable to interact with PCNA (DDB2^PCNA‐^) form clones able to grow without anchorage. Different experimental approaches reveal heterogeneity in cell cycle regulation and drug response within these clones, emphasizing the crucial role of the DDB2‐PCNA interaction in preventing cellular transformation.

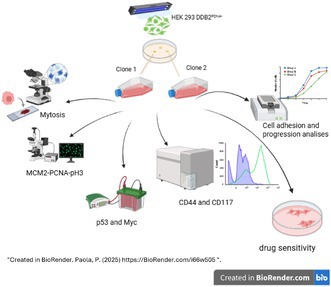

**Abbreviations**
DDB2: DNA damaged binding protein 2
EMT: epithelial‐to‐mesenchimal Transition
MCM2: mini chromosome maintenance 2 protein
NER: nucleotide excision repair
PCNA: proliferating cellular nuclear antigen
RTCA: real‐time cell analyzer

DDB2 protein contains a PCNA interacting protein (PIP)‐box motif in its sequence, which enables direct interaction with PCNA, as demonstrated for other proteins, such as p21 [1]. Mutations in the PIP‐box sequence disrupt this connection determining NER‐deficient response, affecting both GG‐NER and TC‐NER [2, 3]. The loss of DDB2‐PCNA interaction, after UV‐damage induction, favours processes such as proliferative advantage [2] and epithelial‐to‐mesenchymal transition (EMT) activation [4], both of which are involved in tumour initiation and progression.

Recent literature highlights the role of DDB2 not only in tumour growth [5, 6] but also as a predictor of cancer chemoresistance [7] and clinical efficacy of various cancer therapies [8].

In previously published evaluations of the role of DDB2 protein in cancer, either its absence or increased expression level has been considered. In contrast, the impact of DDB2 mutations on cellular transformation and cancer progression remains poorly understood. In particular, its interactions with PCNA, a protein essential for both DNA replication and repair synthesis, may influence modulating these cellular processes.

In a previous study, we have found that cells expressing a DDB2 mutant protein unable to interact with PCNA (DDB2^PCNA‐^) show a delayed DNA repair process [9]. Since defective DNA repair promotes genome instability, which is a typical feature at the onset of cancer development, we sought to further investigate the behaviour of these cells.

In this work, we present data from molecular analyses performed in two cell clones derived from irradiated HEK293 cells expressing DDB2^PCNA‐^ protein. The first aim was to evaluate whether these cells, which are defective in DNA damage response [4], exhibit anchorage‐independent growth, a hallmark of carcinogenesis [10] after UV‐induced damage. The second aim was to investigate whether these resulting cell clones display differences in both molecular and phenotypical behaviour, reflecting the heterogeneity commonly observed in cancer cells.

Applying different experimental approaches, we demonstrate that cells derived from UV‐irradiated DDB2^PCNA‐^ clones show alterations in (a) anchorage‐dependent growth, (b) cell cycle progression and distribution across the different cell cycle phases, (c) cell cycle‐regulating protein expression, (d) number and characteristics of mitoses and (e) drug sensitivity and cell response to treatment. Acquiring all these aspects represents the heterogeneity typical of cellular subclones composing tumour masses, which, over time, show abilities and strategies to overcome the barriers present at various stages of tumour growth.

## Materials and methods

### Cell lines and transfection

HEK293 (Human Embryonic Kidney) cell line was purchased from the European Tissue culture Collection (ECACC) (catalogue code: 85120602). Cell line was cultured in Dulbecco's modified Eagle's medium (DMEM, Sigma) supplemented with 10% fetal bovine serum (Life Technologies‐Gibco Inc., Billings, MT, USA), 2 mm L‐glutamine (Life Technologies‐Gibco), 100 U/mL^−1^ Penicillin and 100 μg/mL^−1^ Streptomycin in a 5% CO_2_ atmosphere.

Cell lines (50% confluent) were stably transfected with DDB2^Wt^ kindly provided by Dr. Q. Wang [12] and the mutated form DDB2^PCNA‐^ using Effectene transfection reagent (Qiagen, Copenhagen, Denmark). DDB2^PCNA‐^ is mutated in the PIP‐box region, as previously described [9]. The clones DDB2^PCNA‐^.1 and DDB2^PCNA‐^.2 were obtained from cells DDB2^PCNA‐^ after clonogenic assay. The cell lines were periodically tested for mycoplasma contamination.

All the experiments were carried out at least three times. Transient transfection of both Wt and mutant DDB2 constructs was also performed in MCF10A and HepG2 cell lines. However, due to the low transfection efficiency in both cell lines, we decided not to continue the experiments.

### Cell growth after UV‐irradiation

Stably transfected cells expressing DDB2^Wt^ or DDB2^PCNA‐^ protein were exposed to UV‐C irradiation (10 J/m^−2^) and then seeded in 96‐well plates (1 × 10^4^ cells/well) to perform a clonogenic assay. The cells were grown for a further 10–15 days with daily medium changes, and one colony of DDB2^Wt^ or DDB2^PCNA‐^ cells was selected and expanded for further experiments.

To evaluate morphological features, the cells were photographed thanks to the inverted microscope (Leitz DM‐IL, Leica, Wetzlar, Germany) at 20× magnification.

For cell proliferation, HEK293 DDB2^Wt^ and DDB2^PCNA‐^ cells (8 × 10^4^) were seeded in 6‐well plates. Cell growth rate was determined by counting the number of cells as a function of the time by using a haemocytometer. DNA synthesis was analysed by measuring bromodeoxyuridine (BrdU) incorporation in cells expressing DDB2. Cells seeded (1 × 10^5^) on coverslips were pulsed for 1 h with 20 μm BrdU to label nuclei in S‐phase, fixed in 2% formaldehyde and then postfixed in 70% ethanol. Fixed cells were washed in PBS, hydrolysed in 2 N HCl for 30 min at r.t. and then neutralized in 0.1 N sodium tetraborate for 15 min. After washing in PBS, samples were incubated for 20 min in PBS containing 0.2% Tween 20 (PBT) and 1% bovine serum albumin. The analysis was performed by immunofluorescence using polyclonal antibody anti‐BrdU (1:100, RRID:AB_11168976; Genetex, Alton Pkwy, Irvine, CA, USA), and then with anti‐rabbit antibody conjugated with Alexa 488 (1:100, RRID: AB_141708; Molecular Probes, Eugene, OR, USA). Then, cells were incubated with Hoechst 33258 dye (0.5 μg/mL^−1^) for 5 min at r.t., and washed in PBS. Slides were mounted in Mowiol (Calbiochem, San Diego, CA, USA), containing 0.25% 1,4‐diazabicyclo‐[2,2,2]‐octane (Aldrich, St. Louis, MO, USA) as antifading agent. Representative fluorescence images were acquired using Leica SP8 Confocal Microscope (STED).

### Isolation of anchorage‐independent cell clones in soft‐agar cultures

HEK293 CTR and stably transfected cells expressing DDB2^Wt^ or DDB2^PCNA‐^ protein were exposed to UV‐C irradiation (10 J/m^−2^). Irradiated and nonirradiated cells (1 × 10^4^) in 500 μL of DMEM (20% FBS) were mixed to 500 μL of 0.33% Bacto Agar (Difco Laboratories, Detroit, MI, USA). Each mixture was poured in culture cell dishes previously prepared with 5 mL of 0.6% Bacto agar in complete DMEM and incubated at 37  °C for 2 weeks. DMEM with 60% FBS amounting to 500 μL was added twice a week to each cell dish. Surviving colonies formed were counted using a 10× magnitude inverted microscope (Leitz DM‐IL, Leica), removed from agar and subcultured. Only a few clones of DDB2 mutant, while none of both wt‐DDB2 and untransfected cells, survived.

Subsequently, we verified the ability of the clones DDB2^PCNA‐^.1, DDB2^PCNA‐^.2 to form colonies on agar, using the same protocol described above. All the experiments were performed on these two clones after further expansion of cell population.

### Morphological analysis

To evaluate morphological features, HEK293 DDB2^PCNA‐^ cells and the clones DDB2^PCNA‐^.1, DDB2^PCNA‐^.2 were seeded (2 × 10^5^) on coverslips contained in 35‐mm cell culture dishes. After 3 days, the samples were stained with May–Grünwald Giemsa using a standard protocol (May–Grünwald, Merck and Giemsa, Carlo Erba). Number of atypical mitoses were counted and photographed under a digital microscope Nikon Eclipse 80i with a camera Nikon Digital Sight DS‐Fi1.

### Cell proliferation and clonogenic efficiency

HEK293 DDB2^PCNA‐^ cells and the clones DDB2^PCNA‐^ .1, DDB2^PCNA‐^ .2 (8 × 10^4^) were plated in 6‐well dishes. Cell growth rate was determined by counting the number of cells as a function of the time by using a haemocytometer.

HEK293 cells (1 × 10^3^) were plated in 60 mm culture dishes and incubated. After a period of 7–10 days, to prevent cell confluence, the colonies were stained with 0.1% Crystal Violet to count by visual scoring. Cells were washed twice in PBS, and the Petri dishes were covered with dye for 20 min under constant stirring. Then, the dye was washed several times with distilled water, and the colonies were air dried and counted. The clonogenic efficiency was calculated as the mean percentage with respect to control cells.

### iCELLigence assay

The iCELLigence System (ACEA Biosciences, San Diego, USA) is a microelectronic biosensor system for cell‐based assays, providing dynamic, real‐time cellular analysis for different tests, and among these, cell adhesion [13].

HEK293 DDB2^PCNA‐^ cells and the clones DDB2^PCNA‐^.1, DDB2^PCNA‐^.2 (10^4^ cells) diluted in 400 μL of medium were loaded in each well. The adhesion was monitored for 6 h.

### Cell cycle analysis

Proliferating cells (1 × 10^6^ cells) were fixed in 70% ethanol. After rehydration, samples were incubated at room temperature (r.t.) for 30 min with propidium iodide (42.2 μg/mL^−1^) in PBS containing 1 mg/mL^−1^ RNase A (Sigma, St. Louis, MO, USA) and then analysed by a Partec Pas II flow cytometer.

### Analysis of DNA synthesis

DNA synthesis was analysed by measuring bromodeoxyuridine (BrdU) incorporation in cells expressing DDB2. Prior to being harvested, cells seeded on coverslips were pulsed for 1 h with 20 μm BrdU to label nuclei in S‐phase, fixed in 2% formaldehyde and then postfixed in 70% ethanol. Fixed cells were washed in PBS, hydrolysed in 2 N HCl for 30 min at r.t. and then neutralized in 0.1 N sodium tetraborate for 15 min. After washing in PBS, samples were incubated for 20 min in PBS containing 0.2% Tween 20 (PBT). The analysis was performed by immunofluorescence using polyclonal antibody anti‐BrdU (1:100, RRID:AB_11168976; Genetex), and then with anti‐rabbit antibody conjugated with Alexa 488 (1:100, RRID: AB_141708; Molecular Probes). Cells were scored for immunofluorescence positivity with a Nikon Eclipse E400 fluorescence microscope. At least 500 cells were counted for each condition, and each experiment was repeated at least three times.

### Immunofluorescence analysis

To analyse MCM2 and PCNA, DDB2 cells grown on coverslips (1.5 × 10^5^) were washed twice with PBS buffer and lysed for 10 min at 4 °C in hypotonic buffer: 10 mm Tris–HCl (pH7.4), 2.5 mm MgCl_2_, 0.1% Nonidet NP‐40, 0.2 mm phenylmethylsulfonyl fluoride (PMSF). Thereafter, coverslips were washed in physiological saline, fixed in 1% formaldehyde (5 min, r.t.) and then postfixed in 70% ethanol [14]. To check phospho‐Histone H3 (p‐H3), the cells grown on coverslips (1.5 × 10^5^) were washed twice with PBS, fixed in 2% formaldehyde for 5 min at r.t. and then postfixed in 70% ethanol. After rehydration in PBS, blocking of unspecific staining was performed in PBT solution. Immunostaining of different proteins involved in the cell cycle was performed by incubation with the following antibodies: anti‐MCM2 (RRID:AB_2141952; BD BioScience, Franklin Lakes, NJ, USA), anti‐PCNA (RRID: AB_2160651; Dako, Carpinteria, CA, USA) and anti‐phospho‐Histone H3 (RRID:AB_310177; Ser10, Upstate, Millipore, Charlottesville, VA, USA). All primary antibodies were used at a 1:100 dilution in PBT. After washing in PBT, the coverslips were incubated for 30 min with anti‐mouse (RRID: AB_141607; Molecular Probes) or anti‐rabbit (RRID: AB_141708; Molecular Probes) antibody conjugated with Alexa 488, diluted 1:200 in PBT. Then, cells were incubated with Hoechst 33258 dye (0.5 μg/mL^−1^) for 5 min at r.t. and washed in PBS. Slides were mounted in Mowiol (Calbiochem), containing 0.25% 1,4‐diazabicyclo‐[2,2,2]‐octane (Aldrich) as antifading agent and visualized on a Nikon Eclipse E400 fluorescence microscope with a 100× objective (NA 1.25). For each condition, at least 500 cells were blindly scored by two independent operators, and each experiment was repeated at least three times. Fluorescence photographs of representative samples were taken with Canon Power Shot A590IS digital camera and processed with the Adobe Photoshop 6.0 software.

### Western blot analysis

To investigate total proteins content, cells were directly lysed in loading buffer (65 mm Tris–HCl, pH 7.5, 1% SDS, 65 mm β‐mercaptoethanol, 10% glycerol, 0.02% bromophenol blue) and boiled. All samples were resolved in SDS/polyacrylamide (10%) gel electrophoresis (SDS/PAGE). For subsequent immunoblotting, proteins were electrotransferred to nitrocellulose membrane (BioRad, Hercules, CA, USA). Membranes were blocked for 1 h in 5% nonfat milk in PBT, probed with primary antibodies, anti‐p‐p53 (Ser15) (Cat# sc‐101 762, RRID:AB_1128570; Santa Cruz Biotechnology, Dallas, USA), anti‐p53 (Cat# sc‐126, RRID:AB_628082; Santa Cruz Biotechnology) and anti‐β‐actin (RRID: AB_476730; Sigma), all diluted 1:1000, and with anti‐c‐Myc 1:750 (BD, biosciences). Membranes were then washed 3 times in PBT and incubated for 30 min at r.t. with anti‐rabbit (Cat# A9169, RRID: AB_258434; Sigma‐Aldrich) and anti‐mouse HRP‐conjugated 1:10 000 (Cat# A9044, RRID: AB_258431; Sigma‐Aldrich, St. Louis, MO, USA) secondary antibodies, according to the first antibody used. The original blots are shown in the supplementary materials. The signal was revealed using enhanced chemiluminescence by Azure c600 Gel Imaging System (Azure Biosystem, Dublin, CA, USA). The quantification of the bands was done compared to the loading control (actin) on the same blot, by the public software ImageJ (https://imagej.nih.gov/ij/ RRID: SCR_003070).

### Flow cytometry analysis of CD44 and CD117

HEK293‐mutated cells and the clones were seeded (7 × 10^5^) in 100 mm cell culture dishes. After two days, cells were harvested and fixed in 2% formaldehyde, and then postfixed in cold 100% ethanol and physiological solution. After rehydration, samples were blocked in PBT buffer containing 1% bovine serum albumin (BSA), and then incubated for 1 h with CD44 (8E2F3) antibody (1:200, RRID:AB_10981151; Thermo Fisher, Waltham, MA, USA) or CD117 (3 μg/1 × 10, RRID:AB_10990055; Thermo Fisher). Past three washes with PBT buffer, the samples were incubated for 30 min and with anti‐mouse Alexa Fluor Plus 488 (1:100, RRID:AB_2633275; Thermo Fisher). The cells were analysed for each sample with a Partec Pas II flow cytometer. In the analysis carried out for each sample, the blank was subtracted.

### Clonogenic efficiency after cisplatin or caffeine treatments

HEK293 DDB2^PCNA‐^ cells and the clones DDB2^PCNA‐^.1, DDB2^PCNA‐^.2 were plated (4 × 10^5^) in 60‐mm culture dishes and after 24 h treated with cisplatin (25 or 50 μm) or caffeine (2.5 or 5 μm) for 1 h. To determine caffeine no‐cytotoxic concentration, we performed an MTT assay; instead, for the cisplatin treatment, we chose already tested doses (Guardamagna et al. 2020). At the end of the treatment, the cells were harvested, resuspended in fresh medium and re‐seeded (1 × 10^3^ in triplicate) in culture dishes. After a period of 7–10 days, to prevent cell confluence, the colonies were stained with 0.1% Crystal Violet to be counted by visual scoring. Cells were washed twice in PBS and the Petri dishes were covered with the dye for 20 min under constant stirring. Then, the dye was washed several times with distilled water and the colonies were air dried and counted. The clonogenic efficiency was calculated as the mean percentage with respect to control cells.

## Results

### Different cellular growth in UV‐irradiated cells

**Fig. 1:**
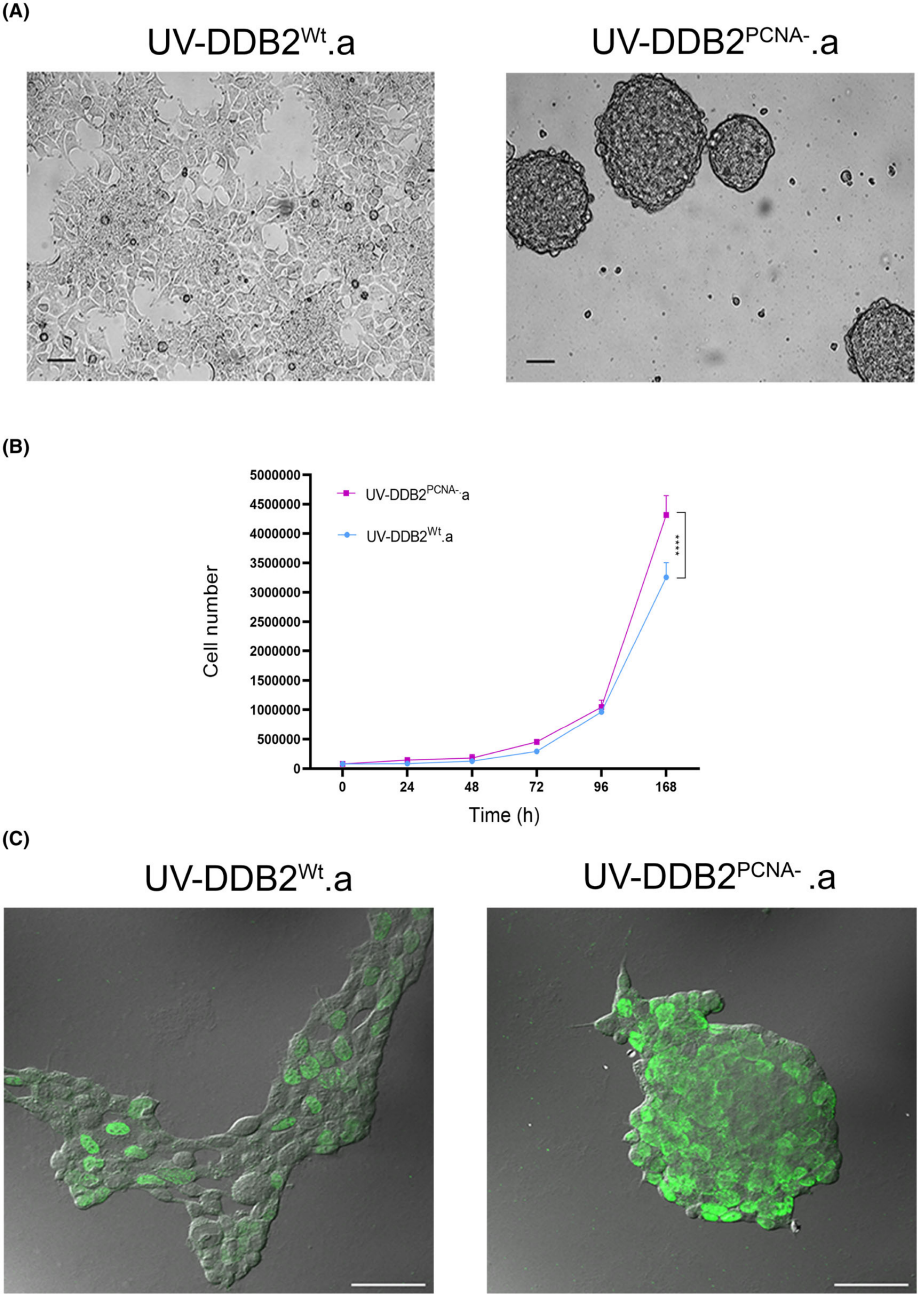
(A) Representative images of selected clones after UV‐irradiation and seeded on Petri dishes. Scale bar: 200 μm. (B) Time course of cell clone growth assessed until 7 days of culture. Results are expressed as mean ± standard deviation. Scale bar: 50 μm. (C) Representative images of BrdU incorporation in UV‐DDB2^Wt^.a and UV‐DDD2^PCNA‐^.a HEK293 clones.

HEK293 cells expressing either wild‐type DDB2 or the DDB2^PCNA‐^ mutated protein were UV‐C irradiated and used for a clonogenic assay. Two clones were selected and referred to as UV‐HEK293 DDB2^Wt^.a and UV‐HEK293 DDB2^PCNA‐^.a, respectively. As shown in Fig. 1A, the UV‐HEK293 DDB2^PCNA‐^.a clone exhibits a markedly altered growth pattern with respect to the UV‐HEK293 DDB2^Wt^.a clone. In fact, mutant cells spontaneously form multicellular three‐dimensional spheroids within 48–72 h, whereas cells expressing the DDB2^Wt^ protein remain adherent. This phenotypic difference is further supported by growth‐curve analysis, which shows an increased number of cells expressing the mutated DDB2 protein (Fig. 1B).

To further confirm these findings, BrdU incorporation was evaluated by confocal microscopy (Fig. 1C). This analysis demonstrated both an increased rate of cell proliferation and the formation of sphere‐like cell aggregates in DDB2^PCNA‐^ expressing cells. Overall, these results indicate that expression of the DDB2‐mutated protein after UV irradiation promotes the development of multicellular aggregates resembling microtumour‐like structures.

### Cells expressing DDB2^PCNA^ ^‐^ protein acquire an anchorage‐independent growth after UV‐C damages

**Fig. 2:**
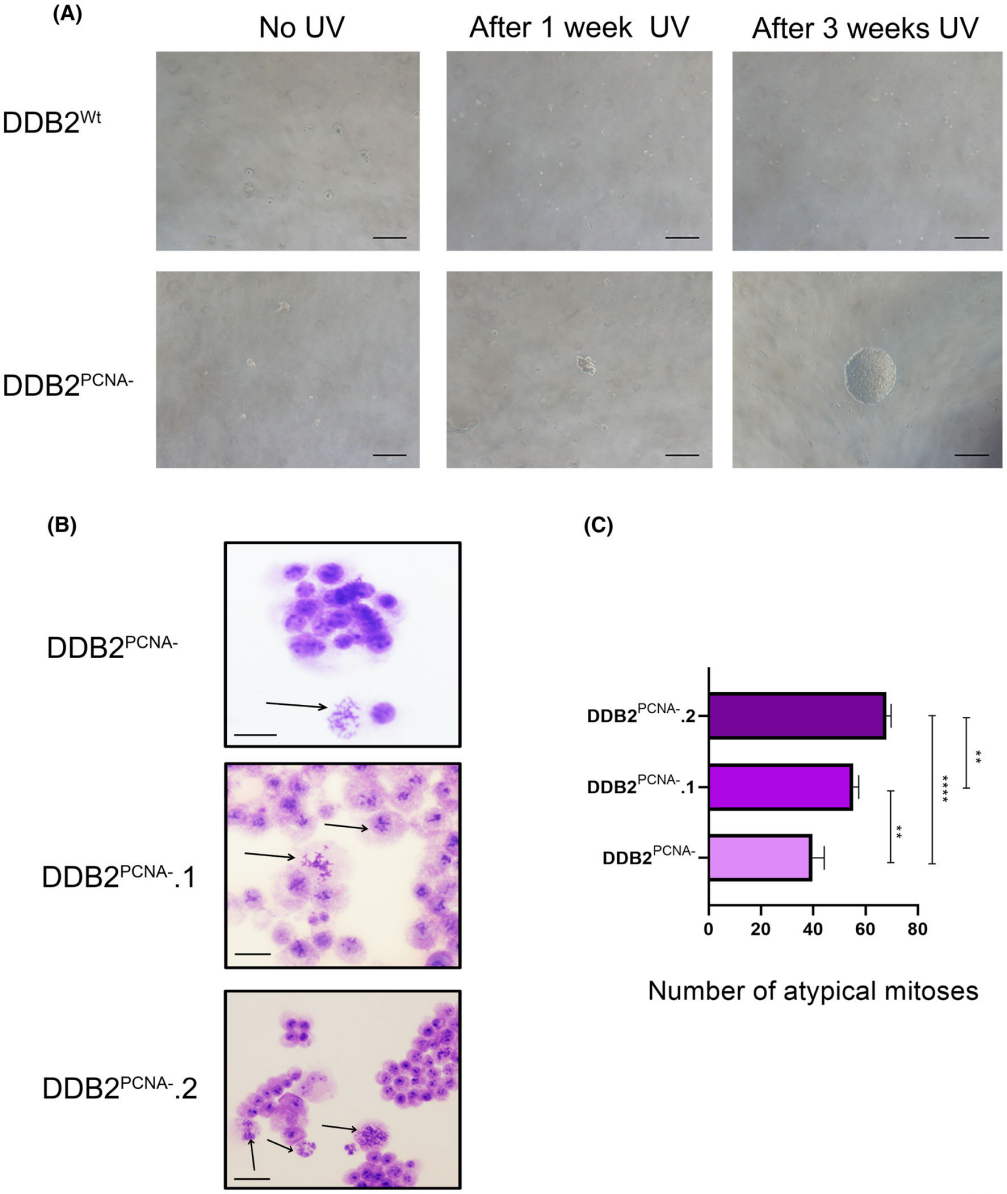
(A) Representative images from soft agar experiments. As shown, 3 weeks of growing after UV determine colonies formation only with cells expressing mutant protein. Scale bar: 200 μm. (B) MCG staining evidences the presence of atypical mitosis in DDD2^PCNA‐^ HEK293 cells and DDD2^PCNA‐^.1 and .2 clones. Scale bar: 50 μm. (C) In this graph, data from atypical mitosis counting are reported. **p < 0.01; ****p < 0.0001. Results are expressed as mean ± standard deviation. Statistical significance was calculated using the one‐way Anova. The Tukey's Multiple Comparison test was used to compare means from several experimental groups. Data are means of at least three independent experiments.

We have previously demonstrated that cells expressing DDB2^PCNA‐^ protein are more resistant to DNA damages caused by UV‐C irradiation and are able to promote EMT [2, 4]. After UV damage, cells were collected and seeded in soft‐agar coated dishes and observed daily for 3 weeks. No cells have grown in the presence of DDB2^Wt^ protein and in untransfected control cells (Fig. 2A and Fig. S1) during all time of observation. Interestingly, as shown in Fig. 2A, cell growth was evident only in the DDB2^PCNA‐^ stable clones. These clones were collected and transferred in new Petri dishes and cultured with complete medium; among those growing faster, we chose two, from now on called DDB2^PCNA‐^.1 and DDB2^PCNA‐^.2.

Coverslips prepared with these clones were stained with May–Grünwald Giemsa to investigate possible changes in cell morphology. As shown in Fig. 2B, we confirm the presence of atypical mitosis in cells expressing DDB2^PCNA‐^ protein and, interestingly, in soft‐agar growing clones we observed an increased frequency of this anomaly; this increase is statistically significant *vs* parental cells expressing the mutant DDB2 protein (Fig. 2C).

Moreover, we have investigated whether the ability to grow in agar, that is without anchoring, is stable and maintained over time by both clones. To this end, we reseeded cells in Petri dishes agar‐coated and the results have shown that both clones retained this ability (Fig. S2).

### DDB2^PCNA^ ^‐^.1 and .2 clones show increased cell proliferation

**Fig. 3:**
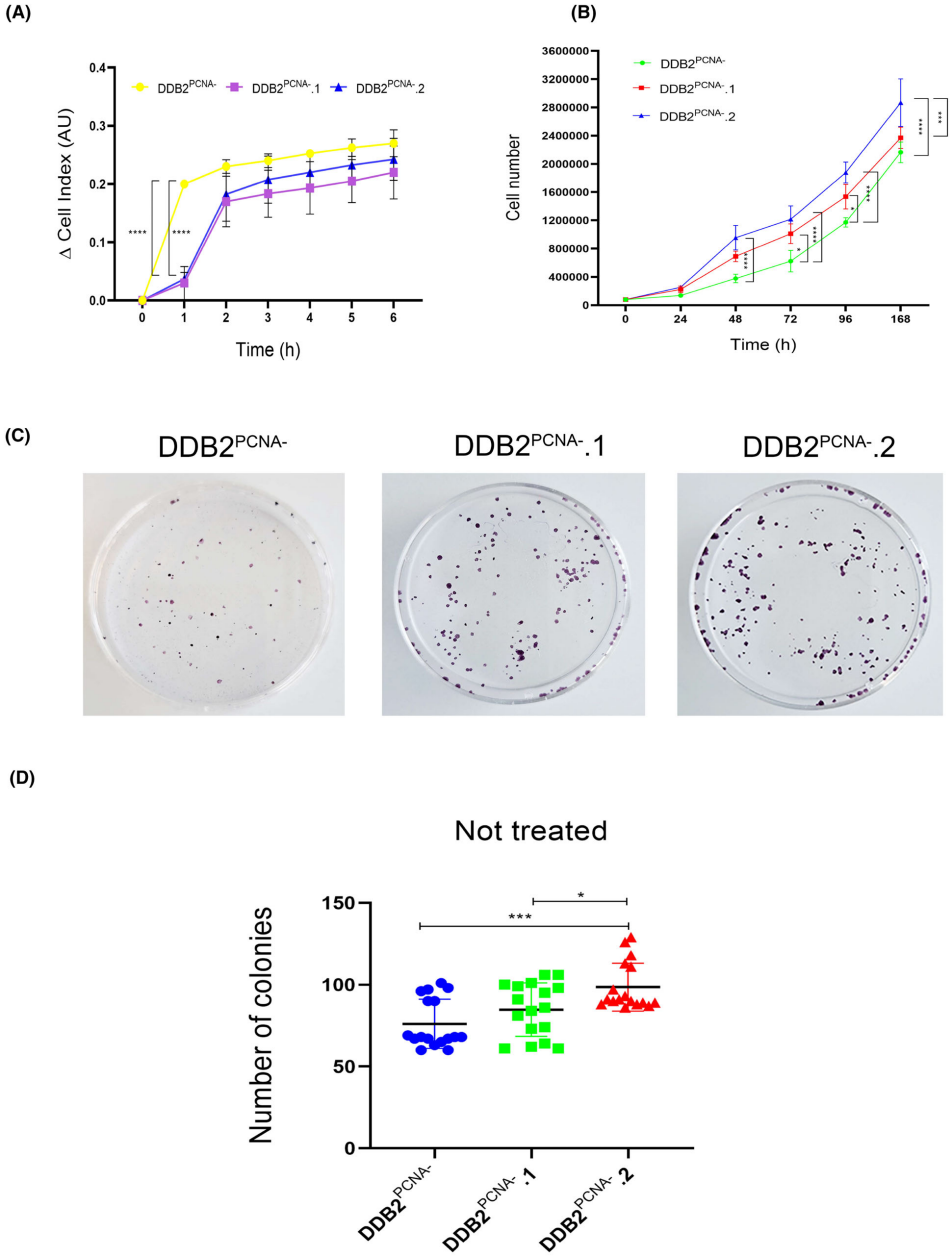
Study of growing rate in parental DDB2^PCNA‐^ cells and DDD2^PCNA‐^.1 and .2 clones using different approaches. (A) Analysis of cellular growth until 7 days (B). Colonies counting formation and statistical analysis (C) *p < 0.05 ***p < 0.001. (D) Time course of cell growth analysed until 6 h with ICELLigence technique. ***p < 0.001. Results are expressed as mean ± standard deviation. Statistical significance was calculated using the one‐way Anova. The Tukey's Multiple Comparison test was used to compare means from several experimental groups. Data are means of at least three independent experiments.

Before studying cell clones' proliferation, we verified their ability to adhere by iCELLigence™ (ACEA Bioscencies, San Diego, USA) real‐time cell analyzer (RTCA) technique. We performed a short time course (until 6 h) in which the cells of the two clones appeared to adhere more slowly than parental cells but only at short times (1 and 2 h); no statistically significant were found at the longer times (Fig. 3A).

In order to verify whether cell proliferation was modified, DDB2^PCNA‐^ cells, DDB2^PCNA‐^.1 and DDB2^PCNA‐^.2 clones were collected and counted daily for 7 days, starting from the day after seeding. As shown in Fig. 3B, DDB2^PCNA‐^.1 and DDB2^PCNA‐^.2 increased their proliferation already at 48 h after seeding, compared to the parental cells, and this difference remained significant until the end of the time course, although at this point the DDB2^PCNA‐^.1 showed a proliferation rate very similar to DDB2^PCNA‐^ cells.

We then determined the cloning efficiency to confirm this different proliferation capability among the cell clones. To this end, HEK293 DDB2^PCNA‐^ cells, DDB2^PCNA‐^.1 and .2 clones were plated and incubated for 10 days to allow colony formation. The results confirmed that both clones formed a higher and significant number of colonies than DDB2^PCNA‐^ cells, especially the DDB2^PCNA‐^.2 (Fig. 3C,D). These data support the results reported above and suggest that the DDB2^PCNA‐^.1 and DDB2^PCNA‐^.2 clones proliferate more rapidly.

**Fig. 4:**
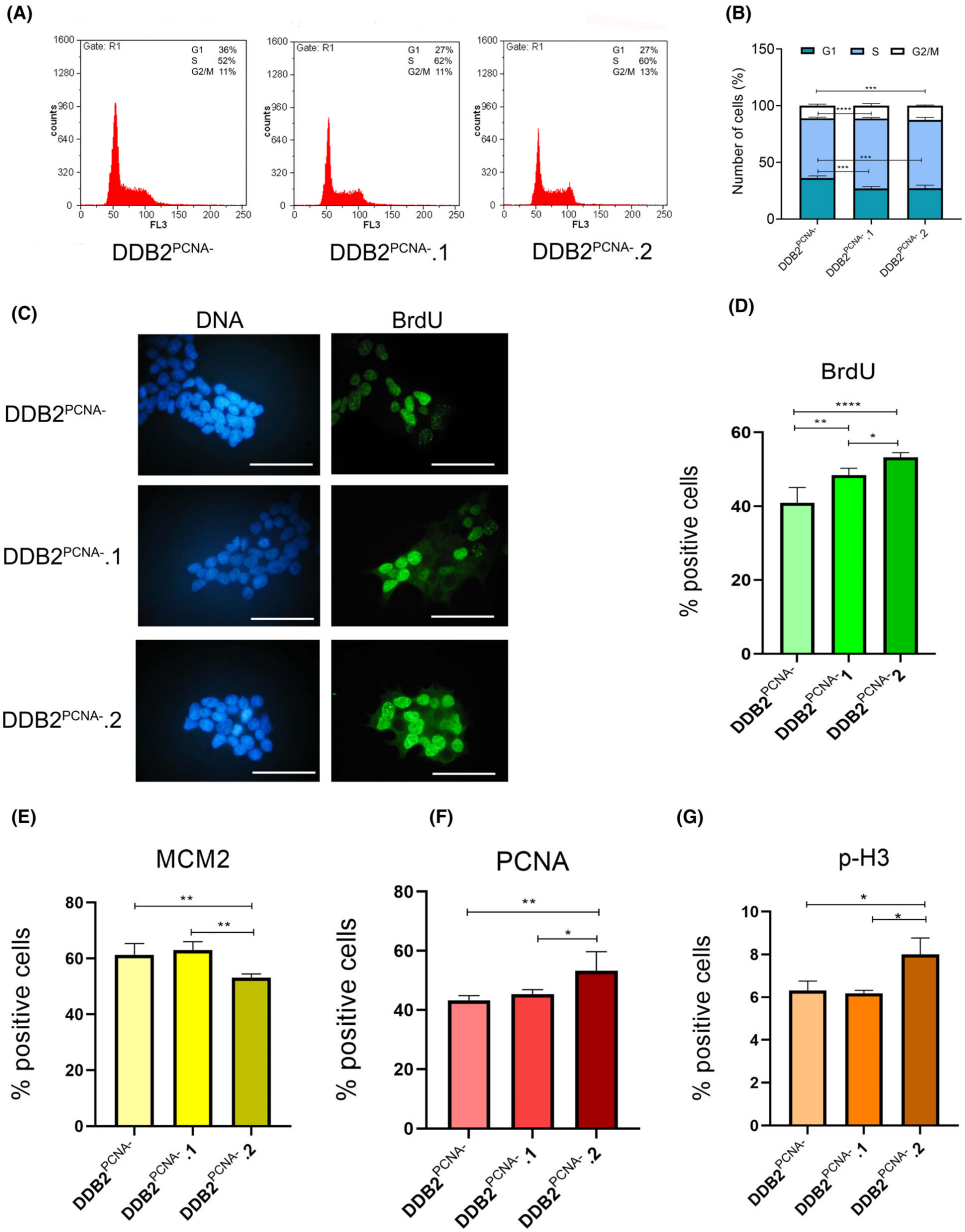
Cytofluorimetric analysis of cell distribution of DDB2^PCNA‐^ cells and DDD2^PCNA‐^.1 and .2 clones in different cell cycle phases (A) and graphical representation (B). ***p < 0.001; ****p < 0.0001. (C) BrdU‐positive cells analysed by immunofluorescence staining and statistical analysis. Scale bar: 50 μm. (D). In (E), (F) and (G) percentage of positive cells for MCM2, PCNA and p‐H3 immunofluorescence staining, respectively, is represented. PCNA and BrdU represent complementary proliferative markers obtained from independent experiments and were not assessed for colocalization. *p < 0.05; **p < 0.01; ****p < 0.0001. Results are expressed as mean ± standard deviation. Statistical significance was calculated using the one‐way Anova. The Tukey's Multiple Comparison test was used to compare means from several experimental groups. Data are means of at least three independent experiments.

To better clarify this difference, cell cycle analysis was carried out by flow cytometry. DDB2^PCNA‐^ cells and cells from DDB2^PCNA‐^.1 and DDB2^PCNA‐^.2 clones were collected and stained with propidium iodide, as reported in the Materials and Methods section. Representative images of DNA profiles are illustrated in Fig. 4A; in both clones a reduction in the number of cells in G1 phase was observed and, at the same time, a marked increase in the number of cells in S phase. The statistical analysis reported in Fig. 4B highlights that these differences are highly significant. To confirm the accumulation of both stable clones in S phase, cells were incubated for 1 h with BrdU, fixed and immunostained by anti‐BrdU antibody and then analysed by immunofluorescence. The results showed a statistically significant increase in the number of BrdU‐stained positive cells in both clones *vs* mutant cells (Fig. 4C,D). A significant difference is also detectable between the data from the two DDB2^PCNA‐^.1 and DDB2^PCNA‐^.2.

### Pre‐replication and replication protein levels are modified in DDB2^PCNA^ ^‐^.1 and .2 clones

To better investigate the DNA replication process, we considered the recruitment onto chromatin of some of the key proteins involved in the process by immunofluorescence analysis. Among these, the protein MCM2, which is a member of the prereplication complex. The recruitment of these proteins on DNA was detected by applying the lysis protocol and immunofluorescence techniques, as reported in the Material and Methods paragraph. Representative images from the obtained results are reported in Supplemental material (Fig. S3). Collected data show that DDB2^PCNA‐^.2 reduces MCM2 recruitment when compared with DDB2^PCNA‐^ and DDB2^PCNA‐^.1 cells (Fig. 4E).

We also investigate PCNA, a pivotal protein involved in S‐phase. In order to study its DNA chromatin‐bound form, coverslips were prepared with cells from both clones and mutant cells, lysed and stained with the specific antibody. The results showed a statistically significant increase in the number of PCNA‐stained‐positive cells in DDB2^PCNA‐^.2 when compared to DDB2^PCNA‐^.1 and mutant cells (Fig. 4F).

Lastly, p‐H3 has been analysed as a specific marker for M phase. The counts carried out show that only DDB2^PCNA‐^.2 has significantly higher levels with respect to both DDB2^PCNA‐^.1 and the mutant cells (Fig. 4G). All together, these results demonstrate that, after UV‐C irradiation, the loss of DDB2‐PCNA interaction further impairs cell cycle regulation.

### c‐Myc and p53 levels are different among the clones

As a way to better understand the molecular mechanisms involved in cell clones' proliferation, we have investigated the expression level of pivotal oncogene (c‐Myc protein) and tumour suppressor genes (p53 protein). It is known that the deregulation of these proteins is associated with human carcinogenesis and their activities were correlated, playing an important role in cell cycle control and proliferation. As reported, c‐Myc protein influences cell cycle progression by regulating the cyclin E/CDK2 complex and, together with E2F protein, determines the assembly of the prereplication complex, assuming a crucial role in the G1‐S transition of the cell cycle.

**Fig. 5:**
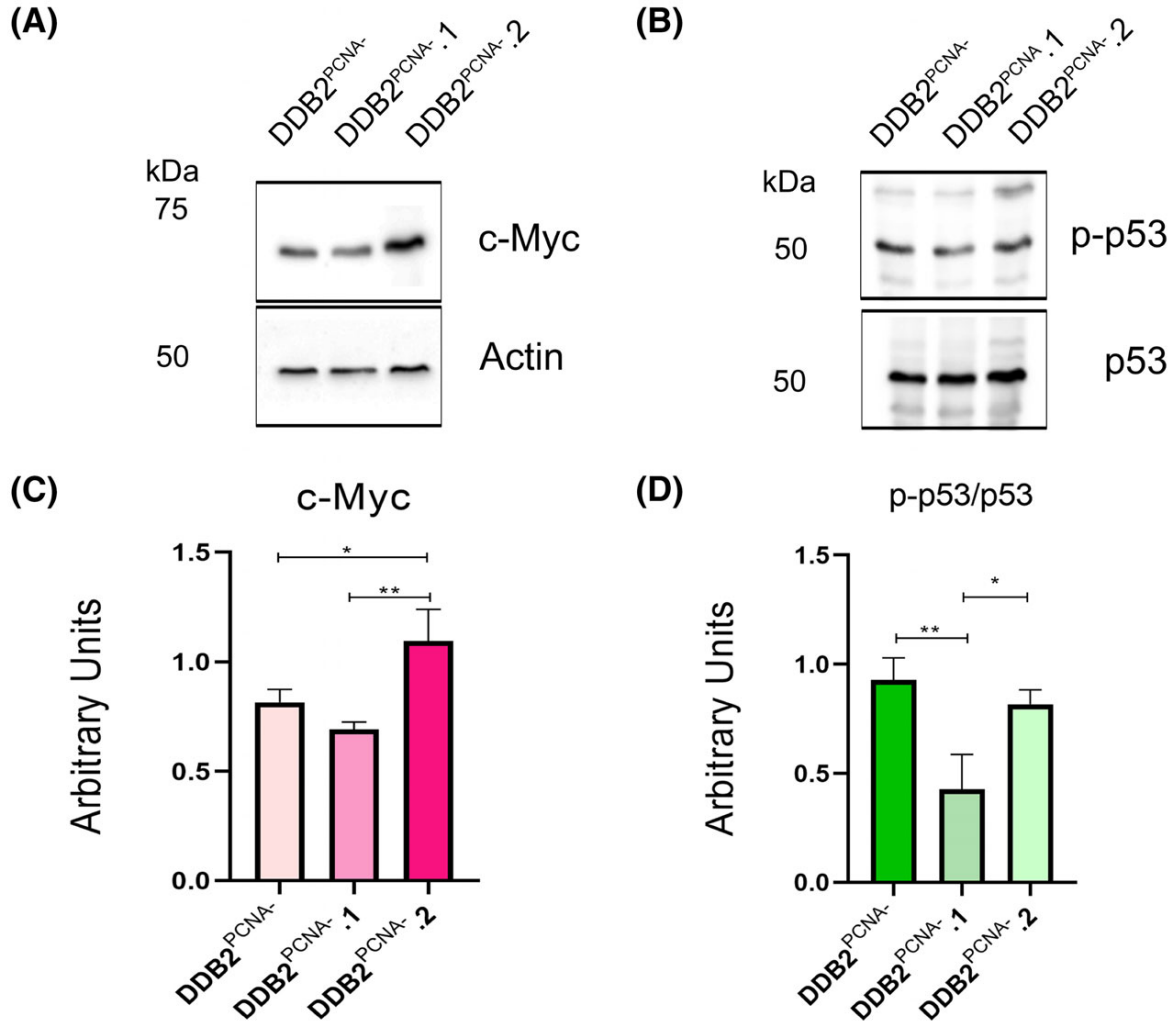
Western blot analysis for oncogene and tumour suppressor proteins. In (A) the expression of c‐Myc is evaluated in DDB2^PCNA‐^ parental cells and DDD2^PCNA‐^.1 and .2 clones. In (B) protein level of p53 active protein is shown. *p < 0.05; **p < 0.01. Results are expressed as mean ± standard deviation. Statistical significance was calculated using the one‐way Anova. The Tukey's Multiple Comparison test was used to compare means from several experimental groups. Data are means of at least three independent experiments.

In this context, we decide to investigate the c‐Myc protein level in mutant cells and the two clones by western blot. In Fig. 5A,C, the results of this analysis demonstrated a significant increase in c‐Myc protein production in clone DDB2^PCNA‐^.2 cells, while in DDB2^PCNA‐^.1, a reduced expression of this oncogene is observed, compared to the DDB2^PCNA‐^ parental line and DDB2^PCNA‐^.2 clone, respectively.

The guardian of the genome, p53 protein has also been analysed considering the total (p53) and hyper‐phosphorylated (p‐p53) protein levels. The ratio p‐p53/p53 represents the amount of active protein and, as shown in Fig. 5B,D, both clones .1 and .2 show reduced protein levels, mainly in the clone DDB2^PCNA‐^.1, thereby suggesting that, in these cells, the control of cell cycle progression is less efficient than in the other clone.

### DDB2^PCNA^ ^‐^.1 and .2 clones exhibit increased levels of CD44 and CD117

**Fig. 6:**
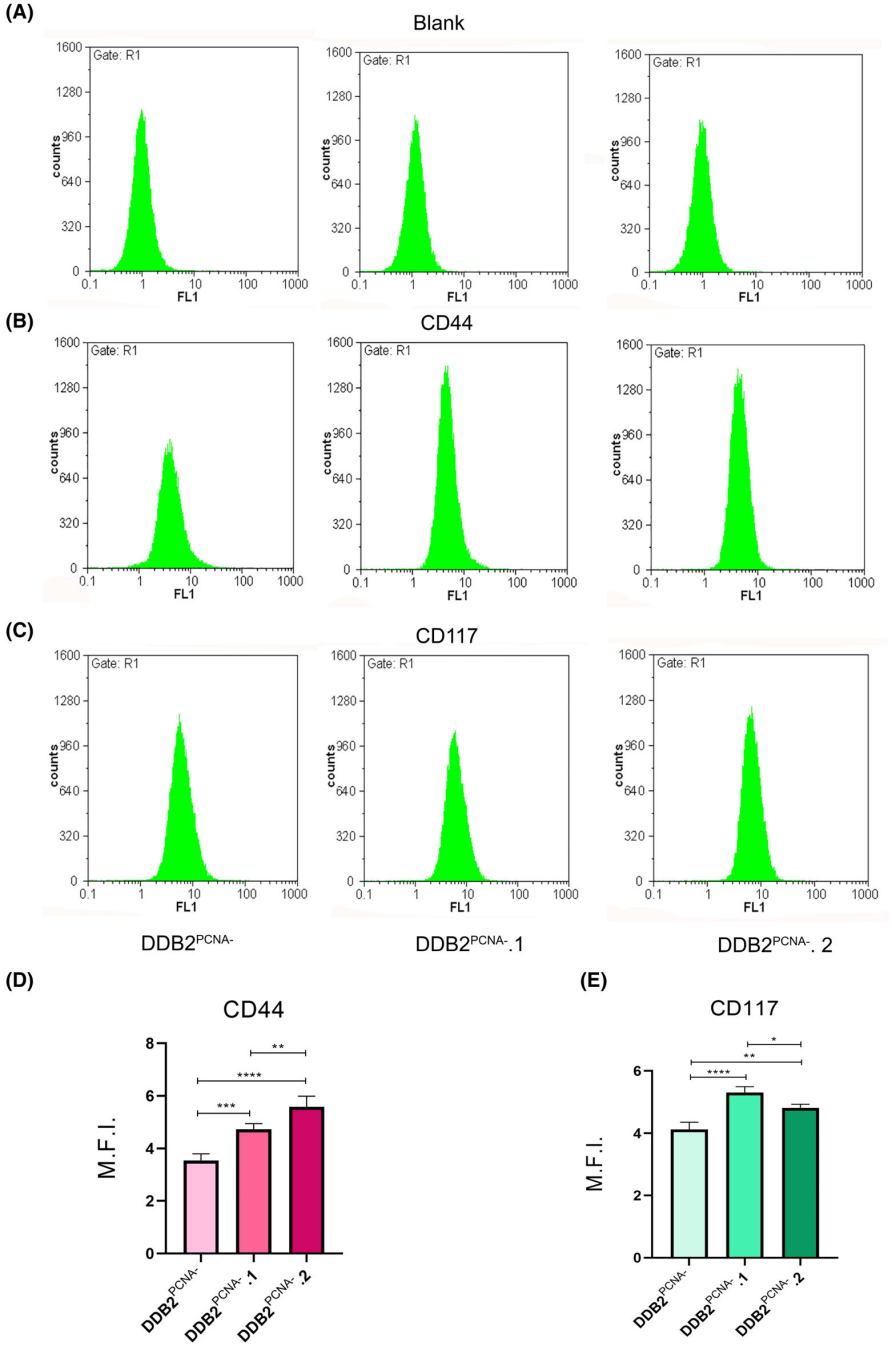
Cytofluorimetric analysis of CD44 and CD117. In (A) cytograms of CD44 and in (B) cytograms of CD117 evaluated in all cell lines. In (C) and (D) statistical analysis from cytofluorimetric data. Data from unlabelled, untreated cells are also shown (Blank). *p < 0.05; **p < 0.01; ***p < 0.001; ****p < 0.0001. Results are expressed as mean ± standard deviation. Statistical significance was calculated using the one‐way Anova. The Tukey's Multiple Comparison test was used to compare means from several experimental groups. Data are means of at least three independent experiments.

To verify whether the DDB2^PCNA‐^ derived clones show properties of stem cells, we have performed cytofluorimetric analysis to investigate CD44 and CD117; these molecules are considered markers of cancer stem cells, and also their expression appears to be correlated in drug resistance [15]. As shown in Fig. 6B,D, the monoparametric analysis of the CD44 antigen demonstrated its increased expression level in both clones compared to cells expressing DDB2^PCNA‐^ protein, and particularly in the DDB2^PCNA‐^.2 clone. Instead, the expression of CD117 is significantly higher in the DDB2^PCNA‐^.1 clone both *vs* DDB2^PCNA‐^ parental cells and the DDB2^PCNA‐^.2 clone. However, although in DDB2^PCNA‐^.2 the CD117 level appeared less than DDB2^PCNA‐^.1, its increase was significant with respect to DDB2^PCNA‐^ cells (Fig. 6C,E).

### DDB2^PCNA^ ^‐^.1 and .2 clones acquire greater resistance to anti‐cancer drugs

Caffeine and cisplatin are two substances studied as anticancer agents, mainly used alone or in combination with other anticancer treatments. Caffeine induces G1 phase cell cycle arrest after DNA damage, activating G1/S checkpoint or in G2 phase blocking the entry into M phase.

**Fig. 7:**
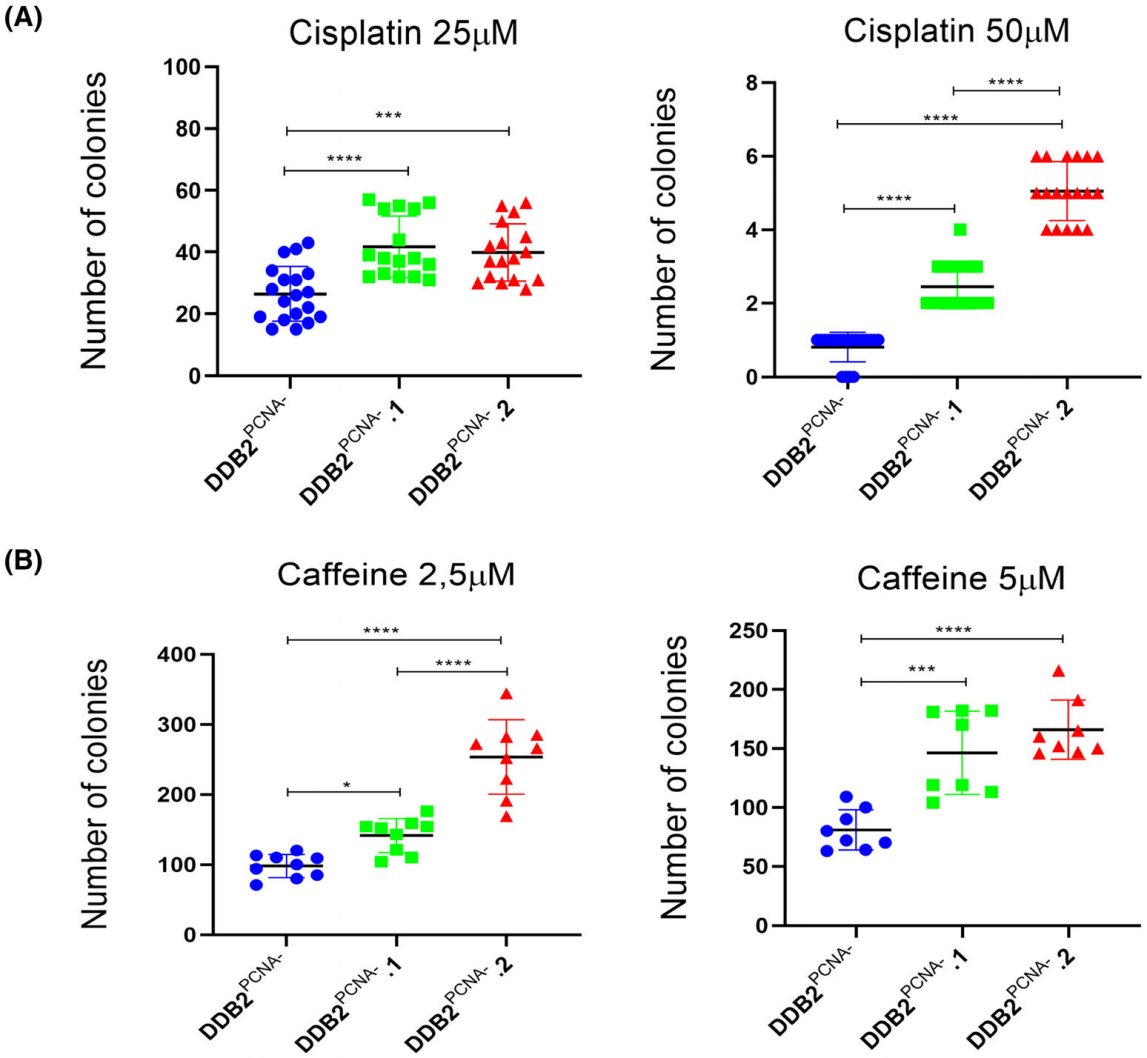
Drug resistance study through number colonies counts in all cell lines. We obtained data from Cisplatin (A) and Caffeine (B) treatment. *p < 0.05; ***p < 0.001; ****p < 0.0001. Results are expressed as mean ± standard deviation. Statistical significance was calculated using the one‐way Anova. The Tukey's Multiple Comparison test was used to compare means from several experimental groups. Data are means of at least three independent experiments.

The caffeine and cisplatin were used, at noncytotoxic concentrations, to treat DDB2^PCNA‐^ cells and DDB2^PCNA‐^.1 and .2 clones. The drug effect on cell growth was assessed with the clonogenic assay (Fig. S4). Cisplatin drastically reduced the ability to form colonies, especially at the 50 μM, compared to the parental line; at this concentration, DDB2^PCNA‐^.2 was more resistant than DDB2^PCNA‐^.1 (Fig. 7A).

Similarly, DDB2^PCNA‐^.1 and .2 showed a significant resistance to caffeine both at 2.5 μm and 5 μm concentrations. In both conditions, clones showed a better clonogenic efficiency compared to the parental cell line; in contrast, no differences were observed between the two clones DDB2^PCNA‐^.1 and .2 at the highest concentration (Fig. 7B).

## Discussion

We previously demonstrated that HEK293 cells expressing DDB2^PCNA‐^, after UV irradiation, acquired MMP‐9 activity and migratory capacity, concomitant with a reduction in E‐cadherin levels [4]. The results prompted us to further investigate the behaviour of these cells. First, we selected two clones, one derived from UV‐irradiated HEK293 cells expressing DDB2^Wt^ protein and one from UV‐irradiated HEK293 expressing DDB2^PCNA‐^ protein. We observed that the latter displays reduced surface adhesion and the ability to self‐assemble into sphere‐like structures. Second, we investigated anchorage‐independent growth, which is one of the most relevant assays for evaluating cancer cell migratory and invasive potential. This experimental end point is typically assessed in *in vitro* models using the soft agar colony formation assay [10]. Using this assay, we have compared irradiated HEK293 cells expressing either DDB2^Wt^ or DDB2^PCNA‐^ proteins and found that only mutant cells were able to grow in this condition. Two of the resulting clones, DDB2^PCNA‐^.1 and DDB2^PCNA‐^.2, were isolated and further expanded to investigate the molecular basis of this newly acquired ability. Confirming our previous studies, we have observed a statistically significant increase in the number of atypical mitoses in the two derived clones, as well as in their proliferation rate and clonogenic efficiency, with DDB2^PCNA‐^.2 clone showing the highest values compared to DDB2^PCNA‐^.1 and, mainly, to the parental mutated cell line. These differences were supported by the analysis of chromatin‐bound levels of MCM2 and PCNA, members of the prereplication and replication complexes, respectively, along with the Ser^10^‐phosphorylated histone H3 (p‐H3), a mitosis marker. It has been previously reported that DDB2 plays a role in the prereplication complex assembly by mediating CDT2 degradation and promoting recruitment of the MCM complex [11], thereby licensing cells to enter S‐phase. Based on our results, DDB2^PCNA‐^.2 cells exhibit reduced MCM2 protein levels and an increased level of PCNA and p‐H3, suggesting a shift of the cell population towards the S ‐ G2/M phases. In contrast, no significant differences were observed between DDB2^PCNA‐^.1 clone and the parental cell line. The modification of cell cycle progression observed in DDB2^PCNA‐^.2 cells may be explained by the high expression of c‐Myc in this clone, which is associated with a reduced expression of p53, both of which are key regulators involved in the cell cycle. In addition, c‐Myc is known to antagonize the activity of the cell cycle inhibitors p21 and p27, either by repressing p21 transcription or by promoting p27 degradation [16].

A functional p53 protein not only represses c‐Myc but also down‐regulates aerobic glycolysis by reducing GLUT1 promoter activity, a glucose transporter overexpressed in many types of human cancer cells [17, 18]. The expression of p53 is also negatively regulated by CD44, a transmembrane glycoprotein highly produced in many human cancers. CD44 is considered a stem cell marker, and its overexpression is associated with EMT and chemoresistance [19]. In our experimental model, CD44 is overexpressed in both isolated clones, likely affecting p53 activity, and supporting our findings on altered cell cycle regulation. In addition, both cell clones showed resistance to Cisplatin and Caffeine, which may be explained by the elevated level of CD44 compared to the parental cell line.

Similarly, the expression of CD117 was higher in both clones than in the parental cell line. CD117 is considered a tumour marker [20], playing a critical role in the regulation of many cellular processes, including proliferation and adhesion [21, 22].

The resistance of the two clones to both Cisplatin and Caffeine, particularly evident in DDB2^PCNA‐^.2 cells at the highest drug concentration, may also be attributed to the inactivation of tumour suppressor p53 [23]. In fact, in our results, a reduction of p53 protein level was observed; consequently, the induction of p21, a crucial step in the cellular response to drug‐induced stress [24], is impaired determining Cisplatin ineffectiveness. This is consistent with previous data showing reduced p21 protein level in DDB2^PCNA‐^ cells [25]. It is well known that p53 activity mediates both cell cycle arrest and apoptosis, processes that are influenced by Caffeine [26]. The antineoplastic effects of Caffeine have been previously demonstrated. More recently, it has been investigated in melanoma, through both *in silico* and *in vitro* approaches, to better understand its mechanism of action in tumour cells [27]. It has been reported that Caffeine modulates cell growth signaling either by directly inhibiting PI‐3 kinase [28] or by activating apoptosis [29]. Our data confirm that Caffeine inhibits cell proliferation in parental DDB2^PCNA‐^ cells, while both isolated clones, particularly DDB2^PCNA‐^.2, displayed significant drug resistance.

The cell clones derived from UV‐irradiated cells expressing DDB2^PCNA‐^ protein show a deregulation of the cell cycle checkpoints and alteration in multiple molecular pathways. Our results obtained on cells growing in soft agar may reflect the dynamic nature of tumour masses, which consist of numerous subclones acquiring distinct behaviours in response to microenvironmental stimuli.

Therefore, the two isolated clones may represent a promising starting point to examine, from both phenotypical and molecular points of view, the differences between them and the parental cell line, and to shed light on the molecular pathways at the basis of the malignant transformation process.

In conclusion, our results indicate that cells expressing a DDB2 mutant protein that is unable to interact with PCNA may acquire, when submitted to additional chemical and physical stressors, molecular features included in the hallmarks of cancer, further supporting the role of DDB2 in preventing malignant transformation.

## Conclusions

In conclusion, this study demonstrates that DDB2‐PCNA binding might play an important role in maintaining genome stability. In our experimental model, the loss of this interaction contributes to cellular heterogeneity at both the molecular and behavioural levels, suggesting its involvement in cancer progression. Additional experiments to rule out a stochastic effect in clonal selection and epigenetic gene regulation will need to be carried out in the future. Furthermore, our findings indicate that the DDB2‐PCNA interaction may influence drug response and resistance, highlighting the need for further investigations. In the future, it will be interesting to explore the role of the tumour microenvironment and dissect the molecular mechanisms underlying cancer heterogeneity.

## Conflict of interest

The authors declare no conflict of interest.

## Author contributions

O.C. designed and supervised the study. P.P., A.T., and M.F. performed experiments and acquired the data. P.G. performed experiments of the revised version of the paper. E.P. performed cytofluorimetric experiments. P.P. analysed the results and made figures and tables. O.C. and L.A.S. wrote the manuscript. L.A.S. and E.P. performed a critical revision. All authors read and approved the final manuscript.

## Supporting information


**Fig. S1.** Representative images from soft agar experiments.
**Fig. S2.** Representative image for clones re‐seeded on soft agar.
**Fig. S3.** Immunofluorescence images for MCM2, PCNA and p‐H3 staining.
**Fig. S4.** Colonies formation after cisplatin (A) and caffeine (B) treatment.

## Data Availability

Data from this study are available from the corresponding author upon reasonable request.
