# Supplementary material for: Anchorage‐independent and faster growth in clonal population from UV‐irradiated NER‐deficient cells
Source: FEBS Open Bio. 2026 Apr 16;16(6):1181–96. doi: 10.1002/2211-5463.70195 (PMC13238810; doi:10.1002/2211-5463.70195)
Supplement: Supplementary file 1 — Fig. S1. Representative images from soft agar experiments. Fig. S2. Representative image for clones re‐seeded on soft agar. Fig. S3. Immunofluorescence images for MCM2, PCNA and p‐H3 staining. Fig. S4. Colonies formation after cisplatin (A) and caffeine (B) treatment. [file FEB4-16-1181-s001.pdf]

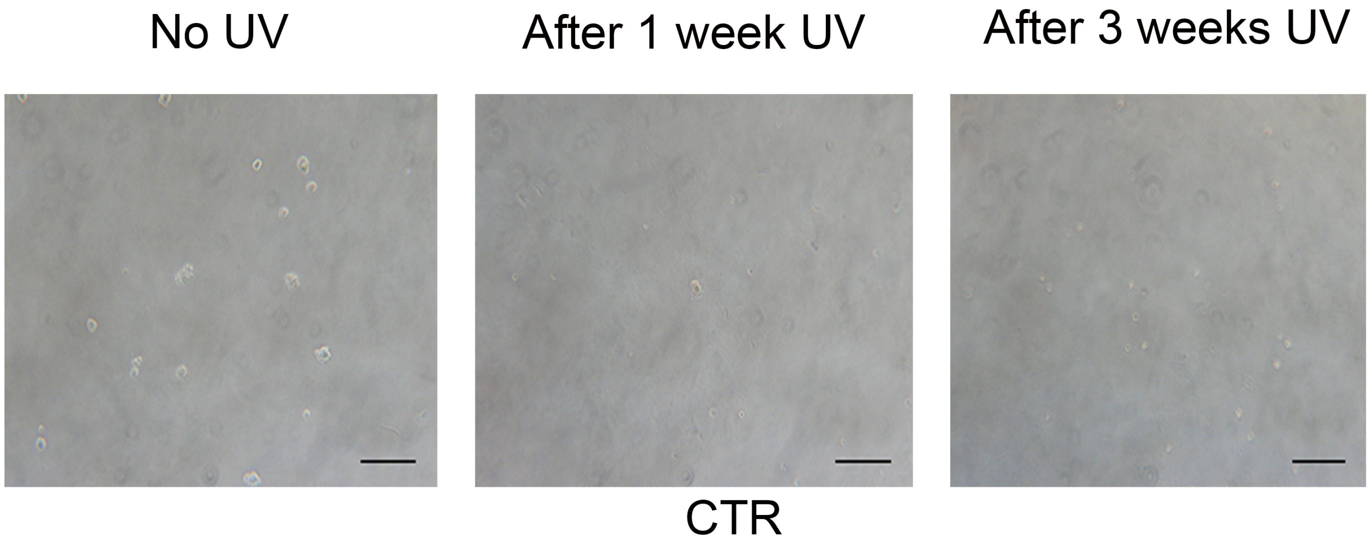

Fig. S1
Representative images from soft agar experiments. As shown, after 3 weeks, no colonies were obtained in HEK293 untransfected control cells. Scale bar: 200 μm.

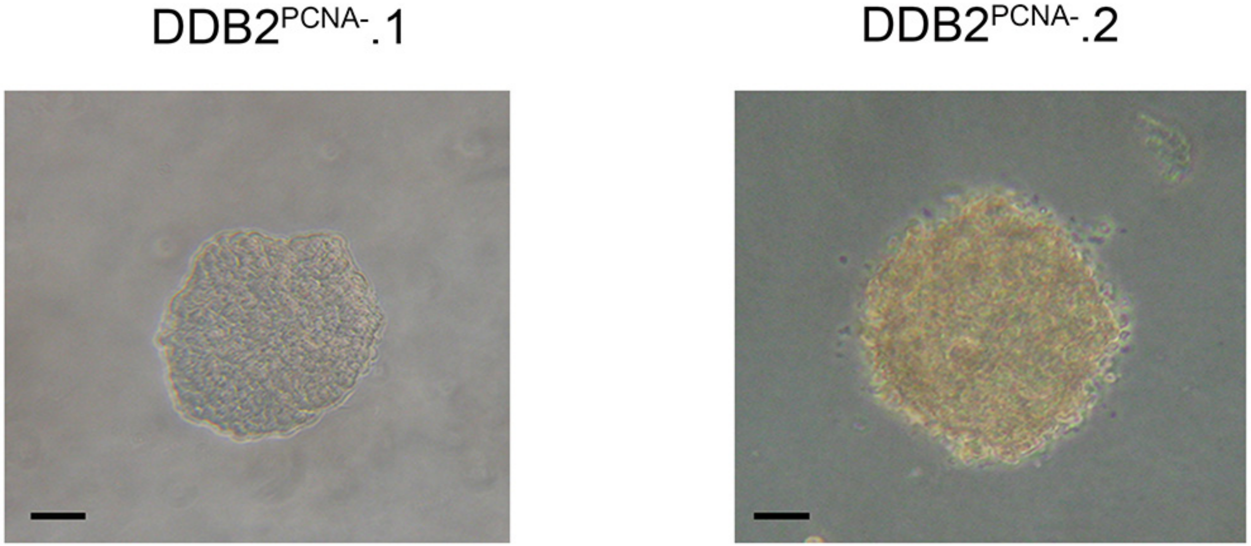

Colonies after 10 days

Fig. S2 Representative image for clones re-seeded on soft agar. Scale bar: 50 μm

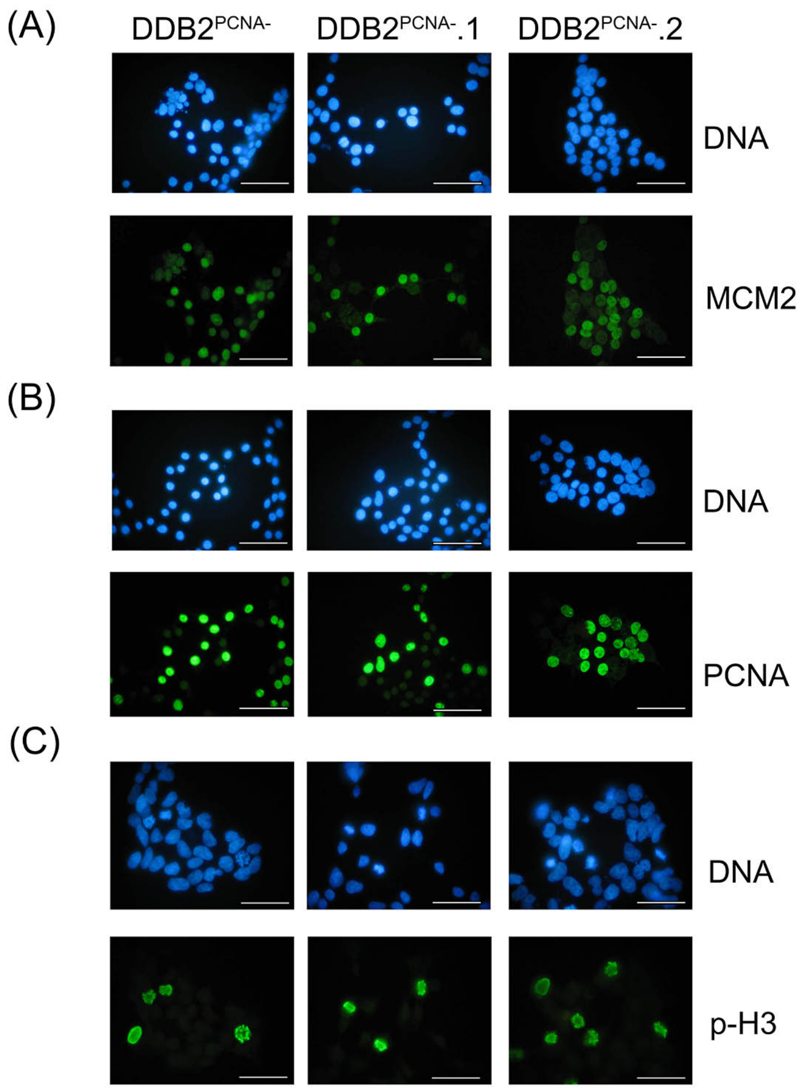

Fig. S3 Immunofluorescence images for MCM2, PCNA and p-H3 staining.
Scale bar: 50 μm

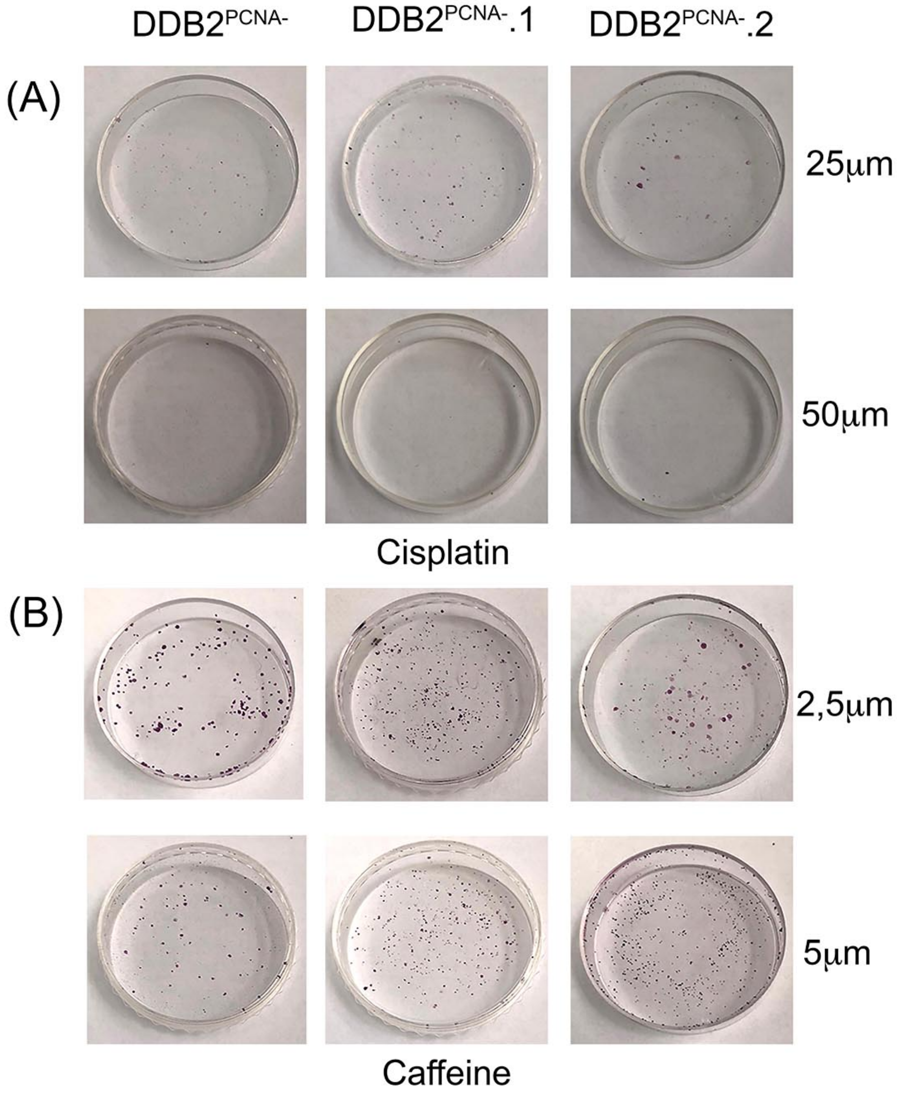

Fig. S4 Colonies formation after Cisplatin (A) and Caffeine (B) treatment
